# Comparison of Unilateral, Asymmetric and Traditional Bilateral Resistance Training in Untrained Women: A Pilot and Proof-of-Concept Study

**DOI:** 10.3390/jfmk11020232

**Published:** 2026-06-08

**Authors:** Atle Hole Saeterbakken, Terese Berger Henriksen, Benedikte Soeviknes Gideonsen, Vidar Andersen, Nicolay Stien, Goran Paulsen, Tom Erik Jorung Solstad

**Affiliations:** 1 Department of Sport, Food and Natural Sciences, Western Norway University of Applied Sciences, 6856 Sogndal, Norway; terese.b.h@hotmail.com (T.B.H.); benediktegideonsen@hotmail.com (B.S.G.); vidar.andersen@hvl.no (V.A.); nicolay.stien@hvl.no (N.S.); tom.erik.jorung.solstad@hvl.no (T.E.J.S.); 2 Department of Physical Performance, Norwegian School of Sport Sciences, 0863 Oslo, Norway; goranp@nih.no; 3 Norwegian Olympic and Paralympic Committee and Confederation of Sports, 0863 Oslo, Norway

**Keywords:** strength training, chest press, seated row, pallof press, 1 RM

## Abstract

**Background:** Both unilateral and asymmetric loading have been used to increase training specificity and create over-load in a target limb to reduce inter-limb asymmetries. The aim of the study was to compare the effects of conducting either unilateral- or asymmetric-loaded resistance training with traditional bilateral resistance training on maximal dynamic and isometric strength in untrained women. **Methods:** Thirty-four women not conducting regular resistance training were randomized into unilateral (UNI), bilateral (BIL) or asymmetric (ASY) upper-body resistance training (2–3·wk^−1^, 10 wk, 24 sessions in total). UNI conducted all exercises unilaterally (one arm at a time), BIL conducted all exercises bilaterally (both arms), and ASY added 10% of the total load to the non-dominant side. Maximal strength was tested in chest press, seated row (1-RM and MVC in both), and pallof press (only MVC). **Results:** At post-test, BIL demonstrated greater bilateral 1 RM strength than ASY (*p* = 0.017, d = 1.25) in chest press, while UNI demonstrated greater 1 RM strength in the dominant side than ASY (*p* = 0.006, d = 1.45). For the other strength tests, no differences were found between groups in chest press (*p* = 0.068–0.481), seated row (*p* = 0.091–0.591) or MVC peak force for both chest press and seated row (*p* > 0.05). All groups demonstrated pre–post improvements for all measurements in chest press (*p* < 0.05) and seated row (*p* < 0.05), but only ASY demonstrated improvements in pallof press on the non-dominant side. **Conclusions:** Compared to traditional bilateral training, unilateral resistance training did not result in similar effects on dynamic or isometric strength. Asymmetric resistance training demonstrated a lower change in chest press strength on the bilateral and dominant sides compared to the other groups.

## 1. Introduction

In many daily activities and limb-dominant sports (e.g., carrying a bag, playing handball, tennis, or football), repeated unilateral loading may contribute to asymmetries in strength development between limbs [1]. Asymmetric strength between limbs may over time increase the risk of injuries [2], which calls for resistance training strategies to counteract the asymmetric strength level. Perhaps the most promising approaches to generate symmetry between limbs are unilateral resistance training [3] or asymmetric loading (i.e., adding more load to one side of the barbell) [4]. Still, the scientific evidence of the different approaches is limited.

Unilateral resistance training refers to conducting resistance exercises using one limb at a time. Unilateral resistance training may be beneficial since it mimics specific movements, such as walking, running, or sprinting [5], includes several muscle groups in the kinetic chain [6,7], induces cross-education [8] or avoids neural interference resulting in bilateral force deficit [9,10]. Notably, the evidence is contradictory in terms of neural interference [11]. More specifically, Häkkinen et al. [12] conducted a 12-week unilateral or bilateral knee extension resistance training intervention among middle-aged and elderly men and women. Both unilateral and bilateral training were efficient in improving maximal strength and muscle activity, but the magnitude of the improvements supports the principle of training specificity (i.e., greater improvements in trained modality than non-trained modality). Gonzalo-Skok et al. [5] examined basketball players conducting unilateral and bilateral squat training over a period of 6 weeks and demonstrated substantially better results in maximal power, change of direction and reduced between-limb asymmetry for the unilateral group than the bilateral group.

Unequal strength between limbs may reduce performance in sports where limb symmetry in strength is crucial (e.g., rowing, weight and power lifting, alpine skiing) [2,11]. Asymmetric loading [4,13] may cause a greater over-load of the weakest limb over time and counteract the imbalance caused by limb-dominated movements. In terms of asymmetric training, the authors are only aware of two cross-sectional studies. Specifically, Saeterbakken et al. [4] examined 5 and 10% asymmetric bench press (i.e., adding 5% and 10% of the total examined bench press performed with 5% and 10% asymmetric loading (i.e., additional load on one side and corresponding de-loading on the other side)) and reported greater muscle activity in the prime movers on the loaded side during 5 RM lifting, whereas the de-loaded side demonstrated muscle activity similar to symmetric loading. Accordingly, Jarosz et al. [13] reported higher muscle activity in prime movers (pectoralis major, triceps brachii, and anterior deltoid) on the loaded side during 2.5%, 5.0%, and 7.5% asymmetric loading compared to the de-loaded side. However, the study by Jarosz et al. [13] was limited by not controlling the lateral movement of the barbell, meaning the participants could move the barbell laterally to counteract the asymmetric loads. Still, the longitudinal training effect of asymmetric loading is uncertain, but theoretically, it may provide greater neuromuscular stress and thereby greater strength and hypertrophic effects. Whether asymmetric loading is a feasible training strategy to reduce inter-limb strength asymmetries, or to allow bilateral resistance training with reduced loading of an injured limb during rehabilitation, remains unclear. One well-established alternative is unilateral resistance training, which may induce cross-education effects [8] and reduce potential neural interference [9,10]; in a meta-analysis, it was shown to improve strength in the nontrained limb by approximately 7.8% [14].

Unilateral resistance training makes it necessary to compensate for the lacking counter-weight compared to bilateral exercises, and unilateral resistance exercises have therefore resulted in greater trunk muscle activation on the contralateral side [6,7,15,16]. Similar trunk muscle responses have been demonstrated in asymmetric testing [4]. Still, the longitudinal effects of conducting unilateral or asymmetric resistance training on trunk muscle strength have yet to be examined.

Traditionally, bilateral exercises are selected as the primary exercises due to their effects on improving strength and power [17,18]. Furthermore, most exercise machines or multi-joint free-weight exercises are conducted bilaterally with symmetric loads. Therefore, bilateral training with symmetric loads may serve as a controlled condition to compare the effectiveness of unilateral or asymmetric training on muscle strength. The aim of the present study was to compare the effects of conducting either unilateral- or asymmetric-loaded resistance training with traditional bilateral symmetric loading on maximal dynamic and isometric upper-body strength in untrained women.

## 2. Materials and Methods

### 2.1. Design

**Figure 1:**
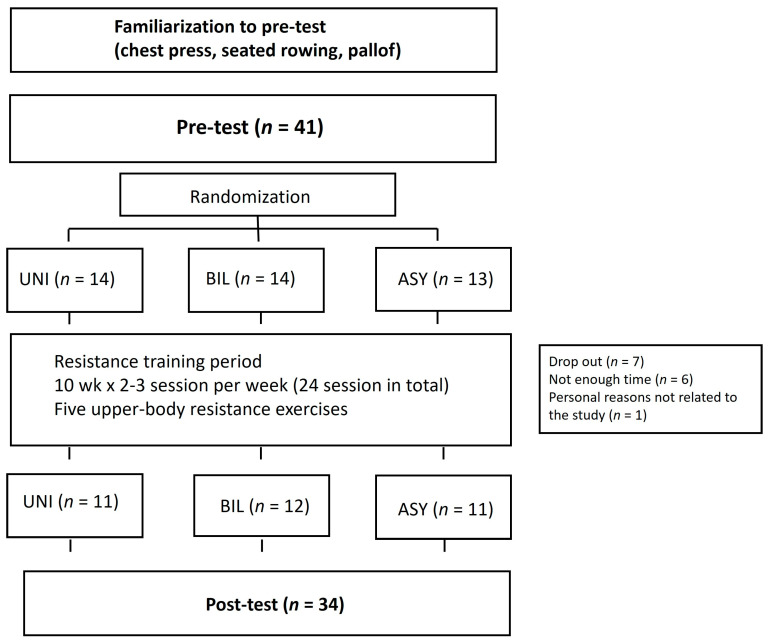
An overview of the study design.

A randomized controlled trail with a within- and between-group design was used to examine the effects of conducting resistance training unilaterally (one limb at a time) (UNI), asymmetrically (greater load on one side) (ASY), and bilaterally with symmetric load (BIL) on bilateral and unilateral dynamic (1-RM) and isometric strength (peak force through MVC) in chest press, seated rowing and pallof press. After one familiarization session, all participants were tested (pre-test) and then randomized into one of three training groups (UNI, ASY or BIL) by drawing lots. After conducting 24 progressive resistance training sessions over a maximum period of 10 weeks (2–3 sessions per week), including five resistance exercises, the participants were tested again (post-test) (Figure 1). All participants reported their dominant arm, defined as the arm preferred to use when throwing a ball.

### 2.2. Participants

To be included, the participants had to be over 18 years old, women, not engaged in resistance training more than once per week, and free from injuries or pain that rendered maximal effort during testing and training impossible; they also had to attend a minimum of 80% of the training sessions. Untrained women are underrepresented in strength training interventions and represent a baseline population with the potential for early-phase adaptation detection and reduced influence of training-induced ceiling effects. The present paper is a pilot study examining proof of concept. Recruiting well-trained athletes to conduct the interventions based on the scarce evidence would likely have been challenging. The participants did not conduct regular resistance training in the last six months (i.e., more than once per week) before being enrolled in the study, but they had previously conducted resistance training randomly. We had no clear inferential goal, and a sample of convenience was included [19] (i.e., as many as we managed to recruit). A total of forty-one women were recruited to participate. During the intervention period, seven participants withdrew (UNI, *n* = 3; BIL, *n* = 2; ASY, *n* = 2) due to reasons not related to the project. For further details of anthropometric measurements of the three training groups, see Table 1.

All participants were informed in writing and orally about the project. Before being enrolled, the participants gave their written consent to participate. The study was approved by the local ethical committee (ref. 22/05321-3) and by the Norwegian Data-protection Compliance Agency (ref. 911469).

### 2.3. Testing Procedures

Before each test or training session, a standardized warm-up protocol was conducted. The warm-up consisted of a 10 min general warm-up (light to moderate intensity, 3–4 on a 1–10 Borg scale) using a treadmill, rowing ellipse, or ergometer cycle. Thereafter, 3 sets (10 reps of 50% of 1 RM, 8 reps of 60 of 1 RM, and 2 reps of 80% of 1 RM) in both seated chest press (Technogym, Pure Row, Cesena, Italy) and seated row (Technogym, Pure Row, Cesena, Italy) were conducted. In the familiarization session, self-reported or estimated 1 RM was used to calculate warm-up loads. In the pre-test and later training sessions, the 1 RM results of the familiarization (pre-testing) and pre-test (training session) were used. A rest of 1–2 min separated each warm-up set.

The testing order was standardized and identical in all test sessions. The order was dynamic chest press, isometric chest press, dynamic seated row, isometric seated row, and pallof (only isometric). For chest press and seated row, unilateral 1 RM of the dominant arm was tested before the non-dominant arm and finally bilaterally. Similarly, isometric unilateral of the dominant arm was tested before the non-dominant arm and finally bilaterally. A 2–3 min rest separated each attempt, and the participants were tested to failure (not able to press or pull the loads to the accepted range of motion; see below for details). The true 1 RM was achieved within 2–4 attempts. For the isometric testing, three attempts were tested for each modality, whereas the best attempt was used in further analyses.

**Figure 2:**
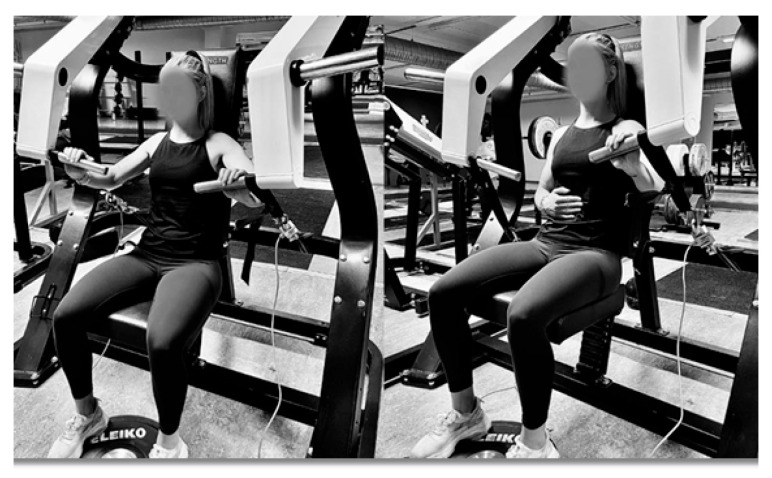
The testing procedures of isometric chest press, bilateral and unilateral.

For the maximal isometric voluntary contraction test (MVC), peak force was used in the analyses. Force output was captured through a force cell (200 Hz, Ergotest Technology AS, Porsgrunn, Norway) attached to the chest press and row machines with non-elastic bands (Figure 2). The length of the bands was the same for all participants (standardized) and corresponded to a 90-degree elbow angle. The participants were instructed to gradually increase force output and maintain maximal force for 5 s. The peak force within this window was identified using commercial software v10.4 (Ergotest Technology AS, Porsgrunn, Norway) and included in further analyses.

### 2.4. Chest Press

**Figure 3:**
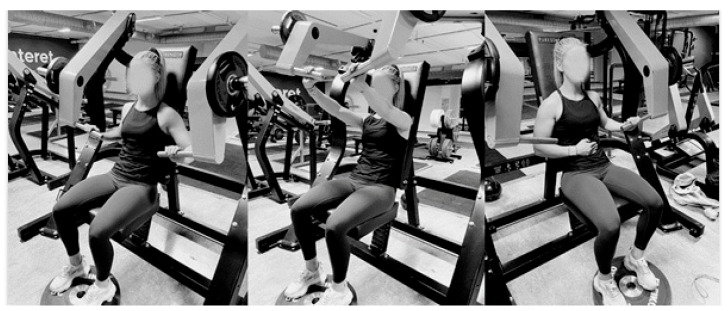
The testing procedures of dynamic chest press, bilateral and unilateral.

In dynamic and isometric testing, the distance between the feet was approximately hip width and in contact with the floor. The seat height was individually adjusted so that the grip handles were at the center of the sternum, and the back, shoulders and head had to be in contact with the seat. The test consisted of only the concentric phase, starting in the lowered position. During dynamic testing, the elbows had to be fully extended before the attempt was accepted as the true 1 RM. During unilateral testing (both dynamic and isometric), the non-testing arm rested on the belly (Figure 3). The ICC (between repetitions of the familiarization test) and CV (familiarization to pre-test) were 0.940–0.964 and 5.9–13.0%.

### 2.5. Seated Row

**Figure 4:**
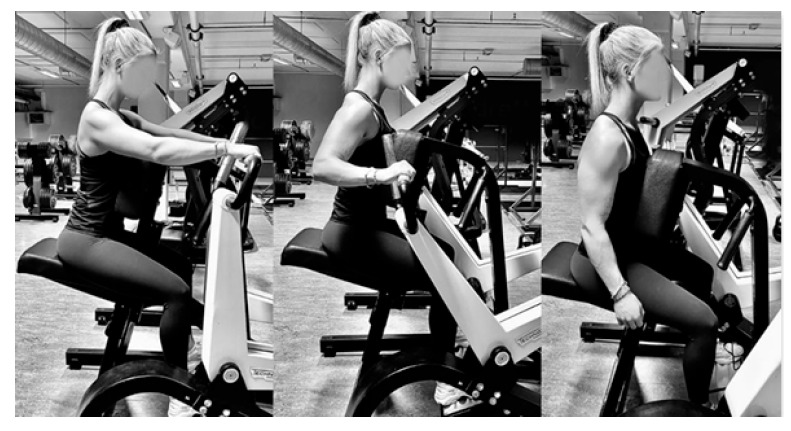
The testing procedures of dynamic seated, row bilateral and unilateral.

During dynamic and isometric testing, the chest had to be in contact with the chest pillow during the entire pull, and the feet had to be in contact with the machine foot pads. The seat height was individually adjusted so that the grip handles were at the center of the sternum when the elbows were extended. The test consisted of only the concentric phase, starting in the lowered position. During dynamic testing, the repetition was approved when the handles were aligned with the chest (visually inspected by the test leader) (Figure 4). During unilateral testing, the non-testing arm was held along the body. The ICC (between repetitions of the familiarization test) and CV (familiarization to pre-test) were 0.891–0.944 and 6.9–12.4%.

### 2.6. Pallof Press

**Figure 5:**
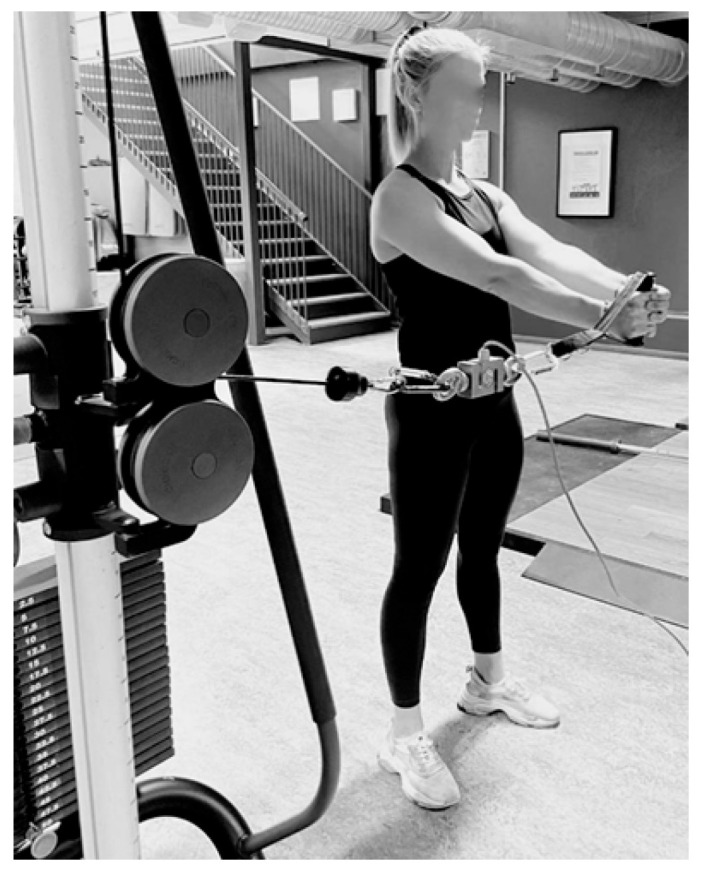
The testing procedures of isometric pallof press.

Pallof press was tested for both the dominant and non-dominant sides, but only isometrically with both arms on the handle. The test was conducted using a cable-cross machine (Technogym, Pure Row, Cesena, Italy). The distance between feet was approximately hip width, and the elbows were fully extended. The heights of the hands corresponded to upper-abdominal height (between sternum and the belly). The cable was adjusted to a similar height, and a force cell (Ergotest Technology AS, Porsgrunn, Norway) was attached between the hand grip and the wire. The weight magazine was set to the maximum as none of the participants were able to lift it (i.e., isometric contraction). The participants were instructed to maintain the hip in a forward position and avoid extensive use of the shoulder muscles (Figure 5). The ICC (between repetitions of the familiarization test) and CV (familiarization to pre-test) were 0.852–0.876 and 8.7–15.2%.

### 2.7. Training Program

Within a period of 10 weeks, 24 training sessions were conducted (i.e., 2–3 sessions per week). The resistance training program was identical for each of the three training groups (i.e., exercises, series, repetitions and intensity (XRM)). The exercises were seated chest press, seated rowing, seated shoulder press, biceps curl and overhead triceps extensions (French press). The exercises were conducted in a fixed order (chest press, rowing, shoulder press, biceps curl, and French press) with only minor deviations when the training equipment was occupied. The first three exercises were conducted using training machines (Technogym, Pure Row, Cesena, Italy) and were identical to the ones used in testing. Of note, the training machines were designed in a way that both arms could be moved independently of each other with individual load magazines for free-weight plates (0.5–25 kg). For the UNI group, one limb was trained before the other. The nontrained limb was rested and placed as it was during 1 RM testing (i.e., no counter-weight), while the BIL group trained both limbs symmetrically and simultaneously with similar loading for both sides (i.e., no asymmetric loading). Dumbbells were used in the biceps and triceps exercises, which were conducted seated (curl) and supine with a 90-degree elbow and glenohumeral flexion angle (French press). The bilateral group conducted all exercises following a traditional approach, where both arms were moved simultaneously. For the unilateral group, one arm was trained at a time, with the nontraining arm resting (i.e., non-load-bearing). The order of which arm started each set was counterbalanced (dominant arm started chest press, non-dominant arm started seated row). Based on previous cross-sectional papers [4,13], 10% asymmetry of the total bilateral load was used, and the load of the de-load side was calculated using the formula total load/two sides + (total load × 10%). To clarify, if the person lifted 100 kg in total (summation of both arms), the loading was 100 kg/2 + (100 kg × 10%) = 60 kg for the loaded side, and 100 kg/2 − (100 kg × 10%) 40 kg for the de-load side. The final total load (sum of both arms) of the last set completed in the last training session was used to calculate and adjust asymmetry. The ASY group therefore reported the lifted loads in the last completed set and received adjusted loads before the next session. The adjusted loads were rounded to the closest 0.5 kg (training machines) or 1 kg (dumbbells).

The first two weeks of resistance training included 3 sets of 10–12 RM for each exercise with a 2–3 min pause between sets and exercises. In weeks 3–5, 3 sets of 8–10 RM were conducted; in weeks 6–8, 4 sets of 6–8 RM were conducted; and in weeks 9–10, 4 sets of 10–12 RM were conducted. If a participant managed to complete the last set with the correct range of repetitions, the load was increased. To adjust the progression, repetition maximum was chosen over % of 1 RM. As the participants were untrained, it was expected that their strength would increase rapidly and substantially throughout the intervention. Consequently, using % of 1-RM would necessitate frequent testing to adjust and correct the training intensity throughout the intervention. Importantly, by using repetition maximum, the load was just adjusted based on the number of repetitions lifted in the previous session. The first four training sessions were conducted with an experienced training instructor who was present in the Fitness studio. For the remaining sessions, the participants trained independently, with the training instructor being present and available to answer any questions. All sessions were logged, where the number of repetitions and loads were reported after each session. The participants were encouraged to maintain normal activity, nutrition and sleep habits during the intervention period, but these things were not controlled for. For all groups, the mean attendance was 23.2 sessions (96%).

### 2.8. Statistical Analysis

All statistical analyses were conducted per-protocol within a frequentist framework to examine the effects of the training intervention. Data are presented as mean ± standard deviation (SD), and statistical significance was set at α = 0.05. Assumptions were assessed prior to interpretation of the analyses. For the paired samples *t*-tests (within-group pre–post analyses), normality of the change scores was examined using the Shapiro–Wilk test. For ANCOVA, normality of residuals was assessed using the Shapiro–Wilk test, and homogeneity of variance was evaluated using Levene’s test. Overall, the data was found to be normally distributed.

To compare potential between-limb strength differences (i.e., dominant and non-dominant limb) at baseline and post-intervention, we performed paired samples *t*-tests with Bonferroni post hoc corrections for multiple group comparisons.

To evaluate between-group differences in training response, analysis of covariance (ANCOVA) was used for each outcome. In these models, the post-intervention value was entered as the dependent variable, group (bilateral, unilateral and asymmetrical) was entered as the fixed factor, and the corresponding pre-intervention value was included as a covariate. For ANCOVA, partial eta squared (η^2^p) was reported as a measure of effect size. Where significant main effects were observed, post hoc pairwise comparisons were conducted with Holm correction for multiple comparisons. To evaluate within-group pre–post changes, paired-samples *t*-tests were conducted for each variable with Bonferroni post hoc corrections for multiple group comparisons.

Effect sizes were interpreted as follows. For Cohen’s d, values of <0.50 were considered trivial, 0.50–1.25 small, 1.25–1.90 moderate, and >2.0 large, in accordance with Rhea [20]. For partial eta squared (ηp^2^), values of 0.01–0.06 were considered small, 0.06–0.14 moderate, and >0.14 large, according to Cohen [21].

All statistical analyses were conducted using JASP v 0.96 (Amsterdam, the Netherlands).

## 3. Results

There were no between-limb differences at baseline (*p* = 0.111–1.000) or post-intervention (*p* = 1.000) in dynamic chest press and seated row in any of the three groups, with the exception of between-limb differences in dynamic chest press (*p* = 0.021, ES = 0.38) in ASY at post-test.

### 3.1. Between Group Comparisons

**Figure 6:**
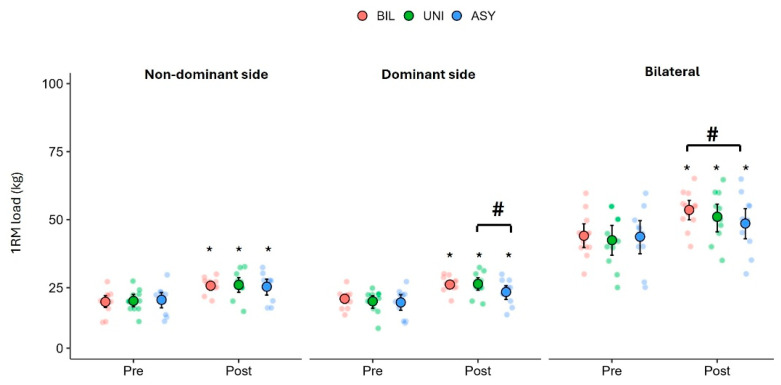
The 1 RM post hoc results for dynamic chest press. # = significant (*p* < 0.05) difference between groups; * = significant (*p* < 0.05) differences pre–post within groups.

For the bilateral dynamic chest press, ANCOVA showed a main effect of group (F(2, 30) = 4.79, *p* = 0.016, η^2^p = 0.242). At post-test, post hoc analyses demonstrated greater strength for BIL compared to ASY (*p* = 0.017, d = 1.25), whereas no statistical difference was observed between ASY and UNI (*p* = 0.068, d = 0.95) or between UNI and BIL (*p* = 0.481, d = 0.299, Figure 6, Table 1).

For the dominant side in chest press, a main effect of group (F(2, 30) = 5.87, *p* = 0.007, η^2^p = 0.281) was observed. At post-test, post hoc analyses demonstrated greater strength for UNI compared to ASY (*p* = 0.006, d = 1.45), while no differences were found between ASY and BIL (*p* = 0.079, d = 0.91) or BIL and UNI (*p* = 0.208, d = 0.539) (Figure 6). No main effects were observed for the non-dominant side in chest press (F(2, 30) = 0.88, *p* = 0.426, η^2^p = 0.055, Table 2).

**Figure 7:**
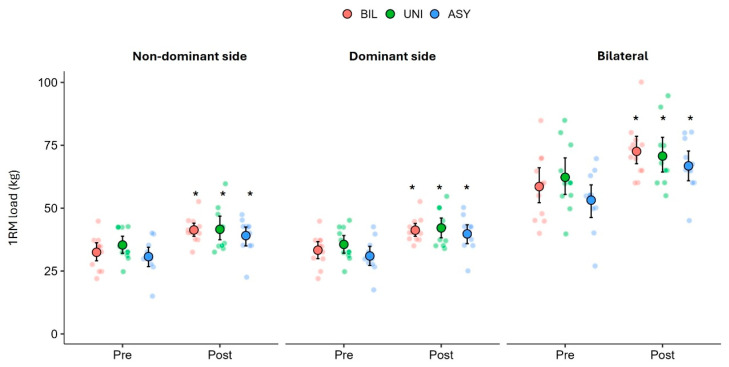
The 1 RM results in dynamic seated row. * = significant (*p* < 0.05) differences pre–post within groups.

For the dynamic seated row exercise (Figure 7, Table 2), no main effects were found for the dominant side (F(2, 30) = 0.54, *p* = 0.591, η^2^p = 0.034), non-dominant side (F(2, 30) = 0.87, *p* = 0.429, η^2^p = 0.055) or bilateral rowing (F(2, 30) = 2.60, *p* = 0.091, η^2^p = 0.148).

For the isometric peak force in chest press, seated row, and pallof press, main effect analysis revealed no significant group effects for any of the dependent variables, with all *p* > 0.05 and effect sizes in the small range (η^2^p = 0.002–0.070, Table 3).

### 3.2. Within Group Comparisons

For UNI, significant pre–post improvements were found for all dynamic chest press and rowing strength measures and in all isometric peak force measurements in chest press and seated row (d = 0.67–2.88). No significant pre–post differences were found in pallof press force. In BIL, the same overall pattern was observed with significant improvements across all dynamic chest press and rowing strength measures and all isometric peak force measures in chest press and seated row (d = 0.75–1.99). No significant changes were found for pallof press. In ASY, significant pre–post improvements were likewise found for all dynamic chest press and rowing strength measures and all isometric peak force measures in chest press and seated row (d = 1.03–3.57). At post-test, ASY was the only group to improve peak force in the pallof press but only on the non-dominant side (d = 0.69). All details are presented in Table 2 and Table 3.

## 4. Discussion

The aim of the present study was to compare the effects of unilateral and asymmetric resistance training with traditional bilateral resistance training on maximal dynamic and isometric upper-body strength in untrained women. The main finding of the study was that BIL demonstrated greater bilateral dynamic strength in chest press than ASY, and UNI demonstrated greater dynamic chest press strength on the dominant side, but not on the non-dominant side, than ASY. For the other dynamic and isometric measurements, no differences were found between groups in any of the three exercises. Notably, within-group analyses demonstrated pre–post improvements for all measurements in chest press and seated row, with only ASY demonstrating improvements in pallof press on the non-dominant side.

The present findings are somewhat surprising and contradictory. For example, one might argue the principle of training specificity [22] as the reason why the BIL group gained greater chest press strength than the ASY group [5]. Still, no difference was observed between the BIL and UNI groups nor in the seated row independent of group comparisons. Perhaps the most reasonable explanation of the findings is related to volume, which has proven significant for both muscle strength [23] and muscle hypotrophy [24]. When calculating the volume (total lifted kg per exercise × set × repetition × session) per subject in the ASY and BIL groups, the BIL group lifted 30% more kg than ASY (76,256 ± 12,835 kg vs. 99,642 ± 13,481 kg). Of note, the reduced volume in ASY was caused by the nature of conducting asymmetric loading, where the loaded limb was trained to failure while the de-loaded limb had approximately 3–5 repetitions in reserve. Given the number of sessions, exercises, sets and repetitions conducted, differences in total volume were expected. Importantly, the loaded limb (dominant limb) in ASY was training similarly to the BIL and UNI, where no differences were observed between groups. Therefore, it is likely that the between-group findings in bilateral chest press are related to total volume, which is supported by the effect sizes (2.37 vs. 1.07). However, these volume differences will only explain the chest press findings, leaving the seated row results difficult to interpret. Especially when both tested exercises were included in the training and demonstrated excellent to acceptable ICC and CV values [25].

In terms of BIL vs. UNI, no differences were observed in the bilateral chest press, in the seated row or in the testing of dominant and non-dominant arms. Comparing the training volume, BIL demonstrated a 5.0% greater volume (99,642 ± 13,481 kg vs. 94,857 ± 24,719 kg) than UNI, which may therefore explain the non-significant difference in muscle strength between the groups. As a potential confounding factor, rapid and non-specific improvements typically observed in untrained individuals may limit the ability to detect minor differences between interventions. Accordingly, caution should be taken when comparing the present findings to studies with a trained population. A strength-trained and sample-sized population may be better able to isolate protocol-specific effects, especially due to low statistical power and the potential of conducting type II errors.

The present findings did not support the bilateral force deficit reported in previous studies [26,27] in the UNI group but instead indicated bilateral facilitation [9] in the seated row. Bilateral deficit appears to be more consistently observed in lower- than upper-body tasks, suggesting a strong task-specific component to the phenomenon [28]. In the seated row, the observed facilitation may be related to the body position and stability demands associated with this exercise. Of note, the authors would emphasize the low statistical power in the present study, making it challenging for this pilot study to detect between-group differences in the UNI and BIL groups. For example, and based on the post-findings for dynamic bilateral chest press, the statistical power was 7.2%. Therefore, the UNI vs. BIL findings were not surprising.

For the unilateral testing of the dominant arm, the difference between ASY and UNI was as expected. More specifically, if a subject in the ASY group lifted 100 kg in total in chest press, a 50% difference in loading between arms would occur (40 kg vs. 60 kg). Since repetition maximum was used to control the intensity, the ability to conduct another repetition in the ASY group was caused by fatigue in the loaded side and not the de-loaded side. The chronic adaptations of lower metabolic and mechanical stress in the dominant arm [4] are most likely the explanation of the difference in 1 RM unilateral chest press strength between UNI and ASY. Therefore, it was surprising that the difference was not significant when comparing ASY and BIL and that no differences were observed in the seated row exercise. A small sample size and large variation in 1 RM strength may explain the non-significant findings (Type II error).

For the bi- and unilateral testing isometric force output in chest press, seated row, and pallof press, no statistical differences between the groups were observed. Isometric testing has typically been reported as “pure” strength or the “ultimate strength capacity” due to the limited intramuscular and intermuscular coordination [29]. Still, due to the task specificity [26,30] and a recent meta-analysis demonstrating more than a two-fold greater effect of dynamic strength than isometric strength after training dynamically [27], the findings of the present study were as expected.

For the within-group analyses, and independent of dynamic or isometric testing conditions, all groups improved dynamic and isometric bilateral and unilateral strength in the dominant and non-dominant arm, with the exception of pallof press. Not surprisingly, the effect sizes were greater (average) in the dynamic testing (small-to-large, d = 1.07–3.44) than the isometric tests (small-to-large, d = 0.77–2.49, Table 2 and Table 3) and comparable to other studies examining subjects who did not conduct resistance training on a regular basis [30]. To the authors’ best knowledge, the present study is the first to examine the training effects of asymmetric resistance training [4,13]. As proof of concept, the asymmetric resistance program improved strength on both the dominant and non-dominant sides, but it reduced the overall training volume and chest press improvement compared to BIL and UNI. Of note, the ASY group was the only group to improve pallof press, but only for the non-dominant side (small effect, d = 0.69). Typically, we would expect greater improvements of the dominant side due to co-contraction of the trunk muscles to counteract the extra loading of the non-dominant side [6,7]. Still, although not statistically different, the non-dominant side demonstrated a small and comparable effect (d = 0.64) to the dominant side. Similar effects between groups may be due to the stability requirement of the training exercises. All exercises were conducted using training machines or with a bench support, which lowers the requirements for using stabilizing muscles during training. Future studies should examine if using more stability-demanding exercises, e.g., free-weights (chest press and rowing) and standing (shoulder press, biceps curl and triceps extension), would lead to greater group differences [6,7].

There are some limitations of the present pilot study that we would like to address. First, the study was conducted without a nontraining control group. Instead, we included the symmetric and bilateral training group to serve as the controlled condition due to the aim of the present study. Therefore, potential isolated training effects or mechanisms cannot be addressed. However, the inclusion of a passive control may be of limited value, as it does not directly inform the comparative efficacy between the treatment conditions. Of note, pilot studies are primarily designed to assess feasibility, refine methodological procedures, and generate preliminary estimates of effect sizes rather than to provide definitive hypothesis testing. Secondly, only healthy women who did not conduct regular resistance training were recruited, and the present findings cannot be generalized to other populations. Of note, early adaptations to resistance training in previously sedentary individuals are often driven by the novelty of the stimulus rather than the specific characteristics of the training protocol. Future studies should examine trained individuals. Thirdly, we only included the upper body. Whether similar findings may occur in the lower body is yet to be examined. Furthermore, we acknowledge that several methodological aspects could have been more rigorously controlled (i.e., more homogeneous sample characteristics and overall experimental control, such as physical activity, nutrition, sleep quality, fatigue and recovery strategies, supplemental intake, and menstrual cycle). However, we would like to emphasize that the use of a randomized three-group experimental design represents a methodological strength, as it provides a robust framework for examining the comparative effects of different training modalities despite potential between-individual differences. Finally, the sample size may have resulted in low statistical power, and we cannot rule out the presence of Type II error. Therefore, we included effect sizes to better interpret the meaningfulness of the findings.

### Practical Implications

The present findings suggest that asymmetric training may be relevant for tasks that require unilateral force production (throwing, kicking, rehabilitation of limbs); however, its application should be combined with bilateral training to achieve more comprehensive adaptations. The findings between unilateral and bilateral resistance training modes follow patterns reported in previous research, supporting the principle of training specificity. Importantly, bilateral training allows for a higher total training volume to be accumulated within a shorter time frame, making it a more time-efficient strategy in applied settings. From a practical perspective, this time efficiency may be particularly advantageous where total training time is limited. Due to the scarce body of evidence, practitioners may consider integrating unilateral, bilateral and asymmetric resistance exercises within training programs to incorporate variation and task-specific adaptations with overall efficiency.

## 5. Conclusions

Unilateral resistance training resulted in similar effects in dynamic or isometric strength compared to traditional bilateral resistance training. Asymmetric resistance training demonstrated lower bilateral and dominant-side chest press strength, while seated row strength was comparable to the other groups at post-test. Further studies should explore trained individuals to explore longitudinal effects of both unilateral and asymmetric loading.

## Figures and Tables

**Table 1:** The characteristics of the participants who completed the protocol and were included in the analyses (mean ± standard deviation).

Group	Age (yr)	Body Height (cm)	Body Mass (kg)
UNI	24.5 ± 4.0	169.1 ± 8.3	69.9 ± 11.3
ASY	24.1 ± 3.5	168.4 ± 6.5	70.2 ± 10.4
BIL	23.3 ± 2.2	167.6 ± 4.8	71.3 ± 8.9

**Table 2:** Overview of the dynamic strength in chest press and seated row.

	Group	Pre	Post	∆ (%)	d (Pre–Post)
Dyn bilateral CP (kg)	BIL	44.1 ± 8.1	53.6 ± 6.8 *	22.9	2.37
	UNI	42.5 ± 9.8	51.1 ± 9.0 *	22.3	2.57
	ASY	43.7 ± 10.6	48.6 ± 12.7 ‡,*	12.7	1.07
Dyn dominant CP (kg)	BIL	20.9 ± 3.2	26.1 ± 2.8 *	26.0	3.44
	UNI	20.0 ± 4.1	26.4 ± 4.2 *	34.8	2.63
	ASY	19.6 ± 5.2	23.4 ±4.5 †,*	22.7	2.18
Dyn non-dominant CP (kg)	BIL	19.8 ± 4.2	25.7 ± 2.9 *	33.4	2.55
	UNI	20.1 ± 4.0	26.0 ± 5.0 *	29.7	2.88
	ASY	20.5 ± 5.1	25.3 ± 4.9 *	25.4	2.37
Dyn bilateral row (kg)	BIL	58.6 ± 13.0	72.6 ± 10.8 *	26.5	2.03
	UNI	62.3 ± 13.3	70.7 ± 12.4 *	15.0	1.54
	ASY	53.2 ± 11.9	66.8 ± 10.3 *	28.5	3.46
Dyn dominant row (kg)	BIL	33.3 ± 6.1	41.3 ± 4.7 *	26.5	1.86
	UNI	35.6 ± 6.3	42.1 ± 7.2 *	18.9	2.16
	ASY	31.0 ± 6.8	39.8 ± 6.8 *	29.9	3.42
Dyn non-dominant row (kg)	BIL	32.5 ± 6.6	41.3 ± 4.9 *	30.4	2.03
	UNI	35.4 ± 6.2	41.6 ± 8.4 *	17.9	1.26
	ASY	30.7 ± 6.9	39.1 ± 6.9 *	29.0	3.57

‡ Significant difference (*p* < 0.05) compared to BIL, † significant difference (*p* < 0.05) compared to UNI, * within group pre–post differences (*p* < 0.05), BIL = bilateral group, UNI = unilateral group, ASY = asymmetric group, and d = Cohen’s d effect size.

**Table 3:** Overview of the isometric strength in chest press, seated row and pallof press.

	Group	Pre	Post	∆ (%)	d (Pre–Post)
Iso bilateral CP peak F (N)	BIL	339 ± 110	426 ± 123 *	28.8	1.55
	UNI	329 ± 65	394 ± 96 *	20.5	0.98
	ASY	319 ± 94	394 ± 102 *	24.8	1.47
Iso dominant CP peak F (N)	BIL	162 ± 47	191 ± 38 *	20.9	1.58
	UNI	149 ± 20	199 ± 40 *	33.8	1.55
	ASY	157 ± 40	180 ± 40 *	16.4	1.03
Iso non-dominant CP peak F (N)	BIL	150 ± 45	178 ± 34 *	22.3	1.50
	UNI	144 ± 21	178 ± 46 *	23.2	1.04
	ASY	146 ± 41	178 ± 45 *	24.0	1.59
Iso bilateral rowing peak F (N)	BIL	501 ± 135	593 ± 112 *	21.3	1.85
	UNI	540 ± 106	605 ± 91 *	14.0	0.76
	ASY	492 ± 117	587 ± 129 *	20.1	2.49
Iso dominant rowing peak F (N)	BIL	294 ± 72	341 ± 66 *	18.3	1.41
	UNI	314 ± 51	349 ± 54 *	12.5	0.77
	ASY	280 ± 68	325 ± 79 *	17.0	1.27
Iso non-dominant rowing peak F (N)	BIL	281 ± 60	324 ± 51 *	17.3	1.02
	UNI	301 ± 42	344 ± 58 *	14.2	1.18
	ASY	283 ± 81	334 ± 95 *	18.6	1.30
Iso pallof press dominant peak F (N)	BIL	105 ± 27	105 ± 24	3.6	0.00
	UNI	106 ± 13	117 ± 22	12.1	0.53
	ASY	102 ± 20	115 ± 31	12.3	0.64
Iso pallof press non-dominant peak F (N)	BIL	104 ± 19	112 ± 27	9.5	0.32
	UNI	108 ± 15	118 ± 16	9.9	0.58
	ASY	108 ± 18	123 ± 32 *	14.0	0.69

* within group pre–post differences (*p* < 0.05), F = force output, N = Newton, BIL = bilateral group, UNI = unilateral group, ASY = asymmetric group, and d = Cohen’s d effect size.

## Data Availability

The original contributions presented in the study are included in this article. Further inquiries can be directed to the corresponding author.
